# The Challenge of Timely, Responsive and Rigorous Ethics Review of Disaster Research: Views of Research Ethics Committee Members

**DOI:** 10.1371/journal.pone.0157142

**Published:** 2016-06-21

**Authors:** Matthew Hunt, Catherine M. Tansey, James Anderson, Renaud F. Boulanger, Lisa Eckenwiler, John Pringle, Lisa Schwartz

**Affiliations:** 1 School of Physical and Occupational Therapy, McGill University, Montreal, Quebec, Canada; 2 Center for Interdisciplinary Research in Rehabilitation, Montreal, Quebec, Canada; 3 Humanitarian Health Ethics Research Group, McGill University, Montreal, Quebec, Canada, McMaster University, Hamilton, Ontario, Canada; 4 Holland Bloorview Kids Rehabilitation Hospital, Toronto, Ontario, Canada; 5 Biomedical Ethics Unit, McGill University, Montreal, Quebec, Canada; 6 Department of Philosophy & Department of Health Administration and Policy, George Mason University, Fairfax, Virginia, United States of America; 7 Department of Clinical Epidemiology and Biostatistics, McMaster University, Hamilton, Ontario, Canada; Renal Division, Peking University First Hospital, CHINA

## Abstract

**Background:**

Research conducted following natural disasters such as earthquakes, floods or hurricanes is crucial for improving relief interventions. Such research, however, poses ethical, methodological and logistical challenges for researchers. Oversight of disaster research also poses challenges for research ethics committees (RECs), in part due to the rapid turnaround needed to initiate research after a disaster. Currently, there is limited knowledge available about how RECs respond to and appraise disaster research. To address this knowledge gap, we investigated the experiences of REC members who had reviewed disaster research conducted in low- or middle-income countries.

**Methods:**

We used interpretive description methodology and conducted in-depth interviews with 15 respondents. Respondents were chairs, members, advisors, or coordinators from 13 RECs, including RECs affiliated with universities, governments, international organizations, a for-profit REC, and an ad hoc committee established during a disaster. Interviews were analyzed inductively using constant comparative techniques.

**Results:**

Through this process, three elements were identified as characterizing effective and high-quality review: timeliness, responsiveness and rigorousness. To ensure timeliness, many RECs rely on adaptations of review procedures for urgent protocols. Respondents emphasized that responsive review requires awareness of and sensitivity to the particularities of disaster settings and disaster research. Rigorous review was linked with providing careful assessment of ethical considerations related to the research, as well as ensuring independence of the review process.

**Conclusion:**

Both the frequency of disasters and the conduct of disaster research are on the rise. Ensuring effective and high quality review of disaster research is crucial, yet challenges, including time pressures for urgent protocols, exist for achieving this goal. Adapting standard REC procedures may be necessary. However, steps should be taken to ensure that ethics review of disaster research remains diligent and thorough.

## Introduction

High-quality research conducted during or in the aftermath of natural disasters such as earthquakes, floods or hurricanes can produce knowledge about the health impact of these events and lead to improvements in the planning and implementation of disaster relief and reconstruction interventions [1–4]. The evidence base for disaster response remains limited, however [5]. For these reasons, increased emphasis is being placed on the importance of conducting research during disasters, as well as during other humanitarian crises [6,7].

The conduct of research in disasters poses significant methodological and logistical difficulties for researchers [8]. For example, clinical trials might be particularly difficult to implement in locales where infrastructure is damaged and public services are in disarray [9]. The context and nature of disaster research also present significant ethical challenges that have been increasingly discussed in the literature [5,10–13]. These ethical challenges arise from the distinctive features of disaster research: populations affected by disaster may be traumatized and highly vulnerable; research activities may impede relief efforts; risks associated with research participation may shift rapidly as post-disaster situations evolve; research protocols may be time-sensitive and need to be implemented quickly after a disaster event; and potential participants may confuse research activities with relief operations.

The research ethics committees (RECs) that are charged with ensuring ethics oversight of disaster research also face challenges, including problems of fit with their standard operating procedures [14]. To date, limited knowledge exists regarding how RECs understand and apply research ethics principles as they review disaster research protocols, and whether and how they modify their procedures for disaster research review. Curry, Walden and Caplan note in relation to research in humanitarian crises that the REC “community has had little collective experience in reviewing and monitoring such protocols” [15].

To address this gap, we undertook a qualitative study to investigate the experiences and perceptions of individual REC members regarding disaster research review. In this paper, we present and discuss results related to elements that REC members identified as important for effective and high quality ethics review of disaster research protocols.

## Methods

We used interpretive description methodology [16] to investigate REC members’ perceptions and experiences in relation to the review of disaster research protocols to be conducted in low- and middle-income countries (LMICs) [17]. Interpretive description methodology aims to identify patterns and linkages amongst individuals who have first-hand experience of a phenomenon of interest, while also accounting for variations between individuals. Interpretive description is grounded in a constructivist and naturalistic approach to inquiry, and supports the development of a coherent conceptual account of a phenomenon [18]. For the purpose of this project, disaster research encompasses studies conducted with human subjects during or following an event such as an earthquake, flood, hurricane, tsunami or typhoon. These events are commonly described as natural disasters, though we acknowledge that the consequences of natural hazards are shaped by social, political and technological factors [19]. We chose to focus on research during disasters in LMICs in order to include considerations related to resource constraints and humanitarian response.

### Recruitment

We used a purposive sampling strategy with the goal of recruiting a diverse set of respondents based on type of REC (e.g. affiliated with a university, government, or non-governmental organization) and geographic location of the REC (e.g. in a high-income country or LMIC). We used four strategies to identify potential interviewees. First, we initiated recruitment through investigator contacts, whereby we approached individuals from our professional networks who had experience reviewing disaster research as part of an REC. Our second strategy was to carry out extensive web searches targeting organizations engaged in disaster research and disaster response (e.g. centers for disaster studies, non-governmental organizations, intergovernmental organizations, and universities) and to review their websites for indications of which RECs reviewed their research activities. Our third strategy involved a review of peer-reviewed disaster research articles with the goal of identifying the RECs that had approved the research protocols. Once we identified an REC through either the second or third approach, we sent an invitation to participate in our study to the chair of the REC or to the REC contact person listed online. Finally, we used a snowball strategy: at the end of each interview, we asked the respondent to suggest other individuals who might be eligible and interested to take part in our study.

### Respondents

We recruited 15 respondents who had experience as chairs, members, advisors, or coordinators of an REC, and who had reviewed disaster research protocols to be conducted in an LMIC. To be included in the study, respondents had to able to participate in an interview in English or French. Characteristics of the respondents and RECs are reported in Table 1. Respondents had experience with a total of 13 RECs that had reviewed at least one disaster study protocol. Two sets of two respondents were members of the same REC. Both of these RECs were amongst those that had the most experience reviewing disaster research protocols and we thus believed that additional insight could be gained by including a second member. One respondent was involved in an ad hoc committee (not a formal REC) that was established during an acute disaster in an LMIC to review research projects because no local ethics review was available. The committee was discontinued once the acute phase of the disaster had passed.

**Table 1:** Characteristics of respondents and RECs.

Sex	Ratio of women to men	4:11
Types of RECs	REC affiliated with a university	6
	REC affiliated with a governmental or international organization	7
	For profit REC	1
	Ad hoc committee established during disaster	1
Location where REC or affiliated organization is based	High-income country	10
	Low- and middle-income country	5
Respondent’s role (one respondent had held two roles)	REC Chair	5
	REC Member	7
	REC Coordinator or advisor	4

### Data collection

We conducted a semi-structured in-depth interview with each respondent by phone, using voice-over-IP technology, or in person. Interviews included open-ended questions and followed an interview guide that we developed based on a review of the literature and our team’s experiences of previous research on related topics [14,20,21]. We tested the interview guide during a pilot interview and subsequently refined it for clarity and comprehensiveness. We also incorporated ideas and insights that emerged from the analysis of earlier interviews into the interview guide as the project progressed [22]. This approach allowed us to test and refine our developing analysis in later interviews. Respondents were asked to discuss the following topics: their overall experience of research ethics review; experiences with disaster research review; processes of review used by their REC, including any alterations for disaster research protocols (e.g. What process was implemented during the disaster? Was there any advanced planning for emergency review of protocols?); how principles or values were included in the review of protocols (e.g. What ethical principles or considerations do you focus on in your review of disaster research?); ethical issues arising for research during disasters (e.g. Are there features of the disaster context or disaster research that you think especially important to address when evaluating disaster research?); and perceptions of ethics guidance and guidelines (e.g. What is your experience of using existing guidance documents or frameworks in reviewing disaster research protocols?). Interviews were conducted from March 2013 to August 2014. All interviews were audiorecorded and transcribed. A team member then reviewed each of the transcripts for accuracy.

### Data analysis

In keeping with interpretive description methodology, we employed an iterative analytic process whereby interviews were coded as soon as the transcripts became available to allow for a responsive relationship between the collection and analysis of data. Transcripts were imported into NVivo software to help organize the analysis process. A synopsis of each interview was then written with the goal of summarizing key points and ideas from the interview. Next, we used constant comparative techniques to develop codes by asking the following questions when reading sections of the transcripts: What is going on here? What is this about? One team member (CT) coded all of the transcripts. The emerging coding structure was refined in discussion with a second team member (MH) who independently coded two transcripts. We then developed broader categories by asking: What patterns and linkages exist in the data? Finally, we developed an overarching analytic structure which communicated core concepts related to the respondents’ views of what elements contributed to effective and high quality ethics review of disaster research.

### Ethics considerations

The study was approved by the Institutional Review Board of the Faculty of Medicine, McGill University. Each respondent read and signed an informed consent form. In this article, we alternated use of male and female pronouns for the respondents (R1 female, R2 male, R3 female, and so on).

## Results

A respondent with extensive experience reviewing research to be conducted during disasters described RECs that are well suited to effectively review these protocols as those that can be "nimble and responsive to the needs of the field, and still deliver high-quality reviews, [in] a timely manner” (R3). The perspective of this respondent echoes the three key elements for effective review of disaster research identified in our analysis: timeliness, responsiveness and rigorousness. The narratives of the respondents suggest that ethics review that is characterized by these attributes will promote the protection of individuals and communities participating in disaster research, without unduly impeding research that has the potential to lead to better understandings of disasters and that can help inform future disaster relief efforts.

Although respondents identified high quality disaster research as a crucial mechanism for improving disaster relief efforts, they also viewed disaster research as raising important ethical issues and posing distinctive and sometimes significant challenges for ethics review, especially when research needed to be implemented rapidly. In response, they described a range of adaptations to REC procedures coupled with a commitment to ensure that the ethical dimensions of disaster research protocols are carefully appraised (summarized in Table 2).

**Table 2:** Characteristics of high quality review and strategies to achieve them.

Characteristic	Description	Challenges	Strategies
Timely	Consideration for the temporal urgency of a disaster protocol	- Standard operating procedures of many RECs are a difficult fit for urgent protocols.	Develop/ adapt procedures for the review of time-sensitive protocols, e.g.
		- Disruption of RECs following a disaster and/or increased number of protocol submissions	1. Convene ad hoc meetings
			2. Conduct review by teleconference or over email
			3. Waive deadlines for protocol submission
			4. Rank protocols by urgency
			5. Have advisors pre-review protocols
			6. Review generic versions of protocols prior to disasters
		- Some protocols, if implemented too early, might lead to re-traumatization or impede relief efforts	- Assess if initiation of research can be delayed and still achieve study objectives
Responsive	Consideration for the realities of conducting research in a disaster, and access to knowledge about the locale where the disaster has occurred	- Few RECs have had the opportunity to develop expertise in reviewing disaster research	- Offer training for REC members regarding the review of disaster research protocols
			- Establish policies and procedures for the review of disaster research
		- Difficult to have knowledge about cultural and social context	- Use of knowledgeable advisors
			- Develop partnerships between RECs
Rigorous	Careful and independent appraisal of ethical considerations related to the research	- Widespread, elevated vulnerability	- Resist the “pressure of urgency”
		- Need for rapid review of urgent protocols	- Provide careful scrutiny of all protocols
		- Protocols may be hastily drafted	
		- Authorities or other actors may seek to influence disaster research or research ethics review, particularly in politically unstable contexts	- Resist external pressures and maintain independence of REC review

### Timely review: “the facts don’t wait for you”

Respondents described the time-sensitive nature of many disaster research protocols as a defining feature of this domain of research, influencing what research is conducted during disasters and how these studies are implemented. Respondents also consistently identified the temporal dimension of disaster research as a primary challenge for its ethics review and suggested timeliness as a key element of effective disaster research review. Timely review was thus seen to encompass consideration for the temporal urgency of a disaster protocol and, when justified, adaptation of review procedures so that urgent protocols are reviewed promptly.

Where research is launched in response to a sudden-onset disaster such as an earthquake or hurricane, researchers may need to initiate their protocols quickly in order to answer research questions pertinent to the acute phase of the disaster response. As described by a respondent who’s REC had reviewed many disaster research protocols, there is a “window of opportunity” (R3) for much of the research carried out following a disaster. Other respondents expressed that “the main issue… is figuring out how to do research fast” (R5) and that reviewers “are sensitive to that in the sense of trying to speed up the protocol’s process of review… because we understand that somebody needs to act rapidly”(R14). These respondents were aware that slow reviews could impede studies requiring activation shortly after disasters, recognising that data from the early phase of the disaster would “never be available again, for obvious reasons” (R9). Several respondents reflected on the lack of congruence between traditional REC review processes and the “demand-driven, short notice” (R4) protocols launched following disasters. A respondent noted that even RECs considered to have a quick turn-around reviewing protocols in normal circumstances might be “really too slow for the facts” during an emergency because “the facts don’t wait for you” (R8).

Respondents described several factors that influenced the speed of disaster research review. In settings where a disaster has occurred, local RECs might be disrupted by the crisis, yet the number of protocols to review might even increase following a disaster. A respondent reported that her REC could not meet for several months after a major disaster in the area. She noted that during that period the REC received fewer protocol submissions than usual. However, in the subsequent months the number of submissions from international researchers increased significantly to levels higher than before the disaster, placing additional burdens on the REC’s infrastructure, which was already stretched due to the disaster.

Decisions of researchers were also noted as influencing the duration of the review process. For example, the chair of a university REC in a high-income country described how some disaster researchers submitted their protocols simultaneously to the university and local RECs to increase the speed of review. He noted that this approach can backfire and lead to “logistical complications” and delays (R14) when RECs make divergent recommendations.

Not all disaster research needs to be implemented in the acute phase of the disaster, and respondents noted that adaptations to facilitate rapid review of such protocols were not needed. They further reported that research following disasters might be implemented prematurely, and this was a possibility that RECs and researchers ought to consider. A respondent gave the example of mental health research, worrying that premature implementation of some protocols might lead to the retraumatization of individuals having just lived through a major crisis. Two respondents also expressed that when implementation of a research protocol could wait (i.e. be started later and still achieve its scientific objectives), it would be preferable to do so in order to minimize the risk of being “disruptive to the relief efforts” (R15) or research participants confusing research activities with relief efforts.

In response to the temporal urgency of some disaster research protocols, respondents discussed a range of approaches to provide timely ethics review. To enhance timeliness, many of their RECs have made ad hoc adaptations to standard procedures or have developed procedures specific to time-sensitive protocols. All of the respondents who were members of RECs that reviewed research to be implemented in the first months following a disaster described tailored procedures or processes for the review of these protocols. The most common deviation from normal procedures was to accelerate the review process for protocols identified as urgent. Strategies included adding additional meetings to review these protocols quickly, conducting protocol review by teleconference or through email exchanges when an in-person meeting was not feasible, waiving deadlines for protocol submission, and prioritizing the review of these studies over less urgent protocols. Four RECs also had a mechanism whereby an advisor who was not a formal member of the REC would review a draft of the protocol and provide rapid feedback prior to its formal submission in order to decrease the likelihood of revisions being necessary. Several of the RECs had used more than one of these strategies.

Two other innovations were described by respondents. First, respondents from two RECs described policies to carry out pre-review of “generic, off-the-shelf” (R3) protocols prior to a disaster occurring, and respondents from two other RECs described initiatives to develop such policies. In this approach, a two-stage review is conducted. First, a generic protocol is reviewed by the REC prior to a disaster occurring and may be given “in-principle” (R6) approval. Once the generic protocol is approved, the second stage involves only a “specific and speedy review of local issues” (R4) where the disaster occurred and how the protocol will be implemented. This approach appears to provide “an ability to respond to a really sudden disaster or a sudden emergency medical condition and make sure we can actually do research in that environment” (R4). Experiences of carrying out such reviews were limited, however, as these policies were new and the RECs using this approach had yet to complete the second stage of the review following a disaster.

A respondent described a different innovative review process. He reported that his REC conducted a two-stage review for especially urgent protocols. Within a few days of the protocol submission, they provided “an opinion on an expedited basis so that work can be initiated” (R12). A full committee review of the protocol then occurred as soon as a meeting could be arranged after which additional recommendations could be given to the researchers.

Overall, respondents viewed innovations and adaptations of review procedures, such as waiving standard deadlines or carrying out the pre-review of generic protocols, as important mechanisms to achieve timeliness.

### Responsive review: “it is very difficult to … understand the situation within that area”

Providing responsive reviews of disaster research requires that REC members have sufficient awareness of and appreciation for the realities of conducting research in a disaster, and that they have access to relevant and up-to-date knowledge about the locale where the disaster has occurred.

Respondents discussed distinctive features of disaster research that are important to consider when reviewing disaster research protocols. For example, they identified that in disaster settings political and social instability is often present, and that risks of research participation are difficult to estimate and are likely to evolve as the disaster situation unfolds. Respondents also spoke about the need to consider issues of security, including the safety of researchers, data collectors, and research participants. In addition, respondents expressed that when reviewing disaster research protocols it was important to acknowledge that standard scientific approaches may need to be adapted due to issues of feasibility during a disaster and that methods may be “circumscribed by the realities of conducting research within disasters” (R4).

Respondents acknowledged, however, that few RECs have opportunities to develop expertise related to disaster research and its review. Developing training or ethics guidance on specific topics arising in the context of disaster research were thus proposed as mechanisms to support REC members seeking knowledge related to disaster research ethics.

In addition to awareness of the distinctive realities of disaster research, responsive review was linked to RECs having access to, and integrating in their review of disaster research protocols, information regarding the cultural and social context where the research would be carried out. This knowledge was described as sometimes difficult to acquire, particularly by respondents who were part of RECs that reviewed protocols to be conducted in other nations. While this concern is not unique to disaster research—for example, it is relevant to international research activities more generally—it was seen as heightened in disaster research due to the elevated vulnerability of participants and the need for rapid ethics review. However, concern regarding a lack of contextual knowledge was not confined to research in distant countries. A respondent from a university REC in an urban setting who reviewed protocols related to an earthquake in a remote, rural region of her country stated that “it is very difficult to, while we are reviewing, to understand the situation within that area” (R13), including cultural and sociopolitical differences. Respondents identified several domains of knowledge that they felt were important for responsive review but were sometimes difficult to access, including gender norms, decision-making structures, and cultural values within a disaster-affected community.

Respondents described approaches that they had used to increase responsiveness when reviewing disaster research protocols. For example, a respondent based at a large research university in a high income country described seeking out individuals within his university who could provide insight about cultural values and norms: To better “understand the situation or the population, we’ll try to find someone … that either comes from that country, has expertise in that country, has expertise with working with this type of population, and we will just get an outside consultation” (R14). Another respondent who was a member of an REC in a high-income country described how his REC had a network of contacts from many regions around the world and would call upon these individuals to gain insight about local contexts.

Increased cooperation and communication between RECs was presented as another strategy to address “gaps in understanding local knowledge, local context, [and] the appropriateness of the study”(R2). A respondent who chaired an REC in a high income country noted that local RECs will “be more familiar with [national research ethics] requirements, [and] will have a better oversight mechanism” (R2). Achieving partnerships of this type is difficult, however, especially given the timelines associated with sudden-onset disasters. Thus, a respondent reported that although collaboration between RECs is valuable, it is unlikely to be effective unless connections existed prior to the disaster. Other respondents noted that collaboration between RECs as a means to increase responsiveness is often impractical due to variability in the accessibility and availability of REC review across locales.

### Rigorous review: resisting the “pressure of urgency” and maintaining independence

Alongside timely and responsive reviews, respondents expressed that disaster research requires rigorous scrutiny to ensure that ethics review provides sufficient protections for individuals and communities participating in research. Such rigorous review of disaster research protocols includes both careful attention to and assessment of ethical considerations related to the research, as well as independence in the review process.

On the whole, respondents saw disaster-affected populations as collectively experiencing increased vulnerability and maintained that disaster research thus requires a high level of ethical scrutiny. In light of widespread vulnerability, a respondent described the protection of vulnerable individuals as the “prime concern” (R6) of disaster ethics review, while a second respondent described that when reviewing disaster research protocols his REC paid particular attention to “the vulnerable nature of [the] population” (R14). This view was formalized in the policies of some RECs. For example, a respondent reported that all disaster research protocols were automatically categorized as requiring the highest level of stringency in ethics review. Many respondents specifically noted that while the speed of review for urgent protocols was increased with the prioritization mechanisms described above, the nature of the scrutiny provided to each protocol was maintained or, indeed, heightened due to the perceived vulnerability of disaster-affected populations. An additional reason for careful scrutiny of disaster research protocols related to the quality of the protocols submitted. Two respondents said that the urgent nature of disaster research limited the time that researchers had to develop their protocols, sometimes leading to more hastily drafted protocols.

As described above, one of the respondents reported that his REC used a two-stage review process for especially urgent protocols in an effort to provide rapid review and ensure that a careful ethical appraisal was carried out. This practice illustrates the challenging balance noted by respondents between disaster research timelines and due rigor in ethics review. In considering potential adaptations of REC review, another respondent expressed that the counterpoint to providing timely review was the need to resist the “pressure of urgency” (R10) and ensure that the review of protocols was rigorous and that ethical issues were given close attention. This balance is reflected by a respondent who described that his REC scrutinized disaster research protocols “in a similar way to other protocols other than that we understood the level of urgency” (R14) and thus made procedural adaptations, such as prioritizing them over other protocols, in order to increase the speed of the review.

Respondents described sources of influence or pressure that arose in the context of disaster research. Several respondents noted that research may be politicized during a disaster and that research, as well as research ethics review, might be used for political purposes or be influenced by political objectives. A respondent described dual challenges for researchers after flooding occurred in a politically tense region where the government sought to control how the disaster was portrayed in reports. He noted that researchers needed to respond to the “urgency of the situation itself and the pressure, the political pressure on researchers in this context” (R10). A second respondent, describing research following an earthquake that had occurred in a neighbouring country, noted that questions are raised for RECs and researchers if “local or your own… politicians get involved and have their own agendas that might affect research or maybe they tell you not to do research and so on” (R9). For example, the chair of another REC described a situation in which her REC experienced pressure to approve a protocol for a vaccine trial after a disaster. The REC did not allow this pressure to influence their assessment of the protocol and sought to “maintain their independence” and “to not politicize the committee” (R1). These respondents emphasized that rigorous review required that RECs maintain their independence and resist outside pressures, while providing careful scrutiny of the ethical aspects of protocols.

### Supporting RECs to review disasters research

Beyond providing ethics review of disaster research protocols that are timely, responsive and rigorous, respondents also discussed several overarching considerations—both at local and international levels—for equipping and supporting RECs to effectively review disaster research protocols. A respondent from an LMIC that had experienced several disasters emphasized the importance of including research and research ethics review as part of disaster planning. She suggested that disaster preparedness should include developing greater capacity and expertise for disaster research and its ethics review in anticipation of future disasters. Several other respondents also emphasized the importance of REC disaster preparedness, including the development of policies, procedures, or templates to support ethics review for research during emergencies. Two respondents also suggested that it would be valuable to establish internationally recognized RECs specialized in the review of disaster research.

When asked about their perceptions of current research ethics guidance, respondents generally did not think that new guidelines were needed to support RECs in the review of disaster research protocols. However, many respondents did identify specific topics where additional guidance, training or support would be beneficial. These topics included methodological innovation in disaster research, the distinction between research and non-research, what to do when local REC review is not available, and adaptations to support rapid review while ensuring due ethical scrutiny of protocols. Respondents expressed that initiatives to develop ethics training or resources related to these topics could help fill gaps in the readiness of members of the wider REC community to review disaster research protocols.

## Discussion

Research ethics oversight is widely viewed as a key process in the protection of individuals and communities who participate in research [23], including in the aftermath of disaster [5]. A key challenge for disaster research review lies in providing rigorous and responsive review, while ensuring its timeliness. Below we discuss several issues associated with timely, responsive and rigorous review of disaster research: procedural innovation and adaptation; proportionality in disaster research review; functioning and capacity of RECs; and advanced planning and ethics guidance.

### Procedural innovation and adaptation

Our respondents described experiences with innovation and adaptation of standard procedures related to the review of disaster research. Some practices are relatively discrete changes, such as waiving deadlines for protocol submission or prioritizing urgent protocols in the queue for review. In other instances, more substantial innovations are being implemented, such as the pre-review of generic protocols. These responses are consistent with growing consensus around the need for procedural innovation for the review of research in public health emergencies [14, 15, 24–26]. Provision for innovative practices has been incorporated in some research ethics guidance. For example, in 2010, a section on research during publicly-declared emergencies was incorporated into the revised Canadian Tri-Council Policy Statement [27]. The guideline directs RECs to carefully assess the need to modify their procedures during a publicly-declared emergency while asserting that “special attention and effort should be given to upholding the core principles of Respect for Persons, Concern for Welfare, and Justice when reviewing the ethics of research to be conducted in emergencies” [27]. Broadly, this study’s findings support judicious adaptation of standard procedures and implementation of procedural innovations for disaster research ethics review, balanced by a commitment to ensuring that the review itself is rigorous and that core ethics principles are upheld. Respondents were supportive of making procedural adaptations for protocols identified as time-sensitive, and they believed that their RECs were able to provide rigorous ethical scrutiny while doing so.

As noted above, the use of generic protocols for pre-review of research to be conducted in public health emergencies is a recent innovation that is garnering attention [24,28]. Experience using pre-assessment of a protocol and subsequent context-specific review is still limited. Respondents in this study did not describe plans to systematically assess the process or outcome of pre-review of generic protocols. Research on the effectiveness and impact of pre-review would thus help to assess the merits of this approach, including inquiry into whether it increases the speed of ethics review relative to other mechanisms aiming to promote timely review. Such research could also consider whether pre-review leads to the development of more robust disaster research protocols as, absent the time pressure of an emergency situation, there would be more time for the considered evaluation of scientific and ethical issues. We also hypothesize that the process of exchange between researchers and RECs involved in generic pre-reviews may contribute to establishing positive and more longitudinal working relationships between disaster researchers and RECs, thus contributing to improved communication and enhanced trust over time. These connections may be particularly important given the potential need for increased interaction between RECs and researchers if risks or other salient features of the disaster were to evolve in ways that raise new ethical considerations after its implementation [29].

### Vulnerability and proportionality in disaster research ethics review

Elevated vulnerability and shifting levels of risk are associated with an important challenge for disaster research ethics review: proportionality of research review stringency to risk. Our respondents described disaster-affected populations as being broadly characterized as experiencing increased vulnerability. As a result, some RECs systematically categorize all disaster research as requiring the most stringent level of review. Several questions are raised by this approach. For example, there is limited evidence that the experience of disaster in and of itself results in exposed individuals having significantly decreased capacities to provide sufficiently informed consent to participate in research [30]. Given the wide variation in risks associated with disaster research protocols and the vulnerabilities experienced by disaster-affected populations, RECs ought to appraise particular protocols in a context sensitive manner. This suggests that it may be unnecessary and even problematic to automatically categorize all individuals affected by disaster as vulnerable persons [31]. Such an assessment will be challenging, however, if REC members have little experience or expertise related to disaster research review [32].

### Functioning and capacity of RECs during disasters

Given the characteristics of disaster research discussed above, ethical challenges unforeseen at the time of the protocol submission might emerge in the course of a study. Few mechanisms currently exist for RECs to be involved in ongoing oversight of disaster research beyond annual renewal requirements, study audits, or formal submission of protocol amendments. Respondents had little experience with ongoing oversight of disaster research and this topic was little discussed by the study respondents, even when they were invited to do so. Fostering ongoing engagement between researchers and RECs could be an important step in helping researchers respond to unforeseen ethical issues arising during the implementation of disaster studies [29].

An added complexity for disaster research in many LMIC settings is the unavailability and lack of capacity for local research ethics review during the chaos that commonly follows a disaster. Local review plays a crucial role in the oversight of research. However, after a major disaster, gathering a quorum of REC members can pose a significant challenge. Those REC members who are not directly harmed by events may be preoccupied with rescue efforts or grieving. Even if a quorum could be safely raised, communications may be difficult in the wake of such an event. As one of the respondents described, RECs may temporarily cease to meet due to the disaster. In such cases, researchers, along with external RECs, need to identify steps required to seek local permission for the research to proceed. In such situations, national or regional authorities might also identify an alternate REC in the country that could be charged with the review of protocols that would normally have been the responsibility of a different REC, thus ensuring availability of REC oversight. Such planning may be particularly important for countries that have limited research ethics infrastructure and few RECs. Ongoing efforts to create networks among RECs, establish more centralized review processes, or mutual recognition of international ethics review [33] might also provide avenues to support ethics review in localities where a disaster has occurred. The rationales motivating these proposals are also consonant with the suggestion made by two respondents that it would be useful to create specialized international RECs with authority and expertise to review disaster research.

Maintaining independence from outside pressure in crisis situations is critical for the legitimacy of RECs and their capacity to fulfill their mandate to provide rigorous review of disaster research. This is one aspect of our findings that has received less attention in the literature. External pressures that threaten the independence of RECs might also occur in situations of political instability, armed conflict or infectious disease outbreak. For example, during an emergency, there might be direct or indirect pressure placed on RECs to allow research to proceed in order to show that something is being done to address the crisis. Research in a disaster setting can also become part of the local “information economy” [34]. Groups such as government agencies might seek to influence REC processes to direct or constrain what types of research can be undertaken in order to control what information becomes available about the crisis, especially if this could lead to criticism of how the situation has been handled by authorities.

### Preparedness and ethics guidance

A range of new resources has become available in the last few years that can also support RECs and disaster researchers. These include the recent World Health Organization training manual on ethics and public health emergencies [35], an ethics framework developed for the Research for Health in Humanitarian Crises initiative [15], and an ethics framework adopted by the Ethics Review Board of Médecins Sans Frontières [24]. At the time of writing, the Council for International Organizations of Medical Sciences is soliciting public feedback on a revision of their guidelines for biomedical research involving human subjects. This revision includes a new guideline on disaster research [36]. Together, these contributions offer valuable resources for disaster research ethics review. Given the perspective of respondents who expressed that formulating new guidelines for disaster research ethics was not necessary, it remains to be seen how these documents will be utilized and whether they will offer sufficient guidance in the areas of uncertainty identified by this study, such as the distinction between research and practice and the use of non-traditional research methods in situations of crises.

Proactive planning can also help RECs prepare for the eventuality that they are called upon to review disaster research protocols in the future. Such disaster preparedness can include the development of policies and procedures to support timely, responsive and rigorous review of disaster research. RECs in all regions of the world can benefit from establishing policies in anticipation of disaster—whether the crisis occurs in their own country or elsewhere. Findings from this study, summarised in Table 2, illustrate challenges and possible innovations for the review of disaster research that should be considered by those drafting REC procedures and policies for the review of disaster research. The findings may also provide inspiration for RECs who are confronted with the need to respond to disaster research protocols that have been submitted to them in the absence of such policies, by suggesting, for example, adaptations to standard procedures that can be implemented to support timely, responsive and rigorous review.

### Avenues for future research

This study examined ethics review of disaster research from the perspective of REC members. In a related project we are also studying perceptions and experiences of researchers who have conducted disaster research. In the future, it would be valuable to examine and compare additional perspectives. For instance, studies that include views from participants and communities involved in research are rare and yet they would help expand the range of perspectives on the topic of disaster research ethics [37]. Other avenues for future research include the assessment of the effectiveness of generic protocol reviews, as well as prospective evaluation and comparison of RECs implementing different adaptations (or not) for urgent disaster research protocols.

### Limitations

Several limitations apply to this study. The objective of this study was to investigate the experiences and perceptions of individual REC members who had reviewed research conducted in an LMIC following a natural disaster such as an earthquake or hurricane. However, many of the respondents also discussed their experiences reviewing research protocols involving toxic spills, epidemics, and armed conflicts. These extrapolations are not surprising given the overlaps between disaster research and research in other public health emergencies, but it did mean that the scope of our inquiry was sometimes wider than originally intended. Second, recruitment of respondents for this study proved challenging. Despite using four recruitment strategies, we had difficulty identifying RECs and REC members with experience reviewing disaster research protocols. This difficulty reflects the limited experience within the international REC community with disaster research review [15]. We ended recruitment with 15 respondents, judging the analytic structure to be sufficiently stable and unlikely to shift significantly with additional interviews.

## Conclusion

In the years to come, RECs around the world will be increasingly called upon to review disaster research. Disaster research is increasing [38], as is the occurrence of disaster events [39]. Disaster research, like research conducted during other public health emergencies [14], is a difficult fit for standard approaches to research ethics review [24], especially when protocols need to be implemented in the days and weeks following a sudden-onset crisis. Ensuring effective and high quality review of disaster research is thus crucial. Through the analysis of the experiences and perceptions of REC members who had participated in the review of disaster research protocols, this study highlights challenges encountered in carrying out reviews of disaster research, documents innovative practices that help RECs meet these challenges, and points to the need for advanced planning and capacity building. This study also identifies attributes of effective and high-quality disaster review: timeliness, responsiveness and rigorousness.
